# A Geospatial Comparison of Distributed Solar Heat and Power in Europe and the US

**DOI:** 10.1371/journal.pone.0112442

**Published:** 2014-12-04

**Authors:** Zack Norwood, Emil Nyholm, Todd Otanicar, Filip Johnsson

**Affiliations:** 1 Energiteknik, Chalmers Tekniska Högskola, Göteborg, Sverige; 2 Department of Mechanical Engineering, University of Tulsa, Oklahoma, United States of America; China University of Mining and Technology, China

## Abstract

The global trends for the rapid growth of distributed solar heat and power in the last decade will likely continue as the levelized cost of production for these technologies continues to decline. To be able to compare the economic potential of solar technologies one must first quantify the types and amount of solar resource that each technology can utilize; second, estimate the technological performance potential based on that resource; and third, compare the costs of each technology across regions. In this analysis, we have performed the first two steps in this process. We use physical and empirically validated models of a total of 8 representative solar system types: non-tracking photovoltaics, 2d-tracking photovoltaics, high concentration photovoltaics, flat-plate thermal, evacuated tube thermal, concentrating trough thermal, concentrating solar combined heat and power, and hybrid concentrating photovoltaic/thermal. These models are integrated into a simulation that uses typical meteorological year weather data to create a yearly time series of heat and electricity production for each system over 12,846 locations in Europe and 1,020 locations in the United States. Through this simulation, systems composed of various permutations of collector-types and technologies can be compared geospatially and temporally in terms of their typical production in each location. For example, we see that silicon solar cells show a significant advantage in yearly electricity production over thin-film cells in the colder climatic regions, but that advantage is lessened in regions that have high average irradiance. In general, the results lead to the conclusion that comparing solar technologies across technology classes simply on cost per peak watt, as is usually done, misses these often significant regional differences in annual performance. These results have implications for both solar power development and energy systems modeling of future pathways of the electricity system.

## Introduction

Comparison, through computer modeling and simulation, of solar power technologies is not a new field. The work of Quaschning [1], for example, analyzed centralized solar thermal electric and PV technologies, and concluded based on analysis of 64 sites that in areas of high solar irradiance thermal-electric technologies were economically favorable to PV (even with cost projections to today) but vice-versa in areas of lower solar irradiance. Multiple renewable technologies have also been compared from a resource-technology perspective by studies such as Jacobson et al. [2] There is even work to quantify the potential of PV technologies over large GIS data sets for both the European continent [3] and North American regions [4]. Extensive modeling of solar technologies to predict efficiency based on fundamental electric and thermodynamic principles has also been published extensively, such as in the work of Kalogirou [5], [6], and Jiang et al. [7], amongst many others. What the body of literature lacks, however, and what we try to contribute with this work, is comprehensive methods and results combining GIS modeling with appropriate physical and empirically verified models of a representative group of current and future cross-sector solar technologies. Additionally, analysis of these technologies based on typical weather data, optimized array tilts, and engineering first principles across such a large geospatial data set (12000+ points in Europe and 1000+ in the US), has not to our knowledge been undertaken. Lastly, the cross-disciplinary nature of this study focusing on *distributed* electric-only, thermal-only, and combined heat and power systems sets this study apart from the field.

## Background

Solar energy is harnessed today, in practice, by two main types of technology: thermal systems collect the light from the sun and either use the thermal energy directly or convert that thermal energy to electricity through a heat engine, whereas photovoltaic (PV) systems convert the photons from sunlight directly into electricity in a semiconductor device. Solar collectors are usually more efficient at converting photons into heat than electricity. Even though the photovoltaic process is more direct, the overall efficiency (percent of sunlight incident that is converted to electricity) of commercial solar thermal-electric and photovoltaic systems fall in similar ranges (10–30%), with the high end of this range reached in both exemplary high concentration PV (HCPV) and concentrating solar power (CSP) systems.

All solar power technologies collect electromagnetic radiation from the sun, but if a system optically concentrates the light (e.g. CSP) it collects primarily the direct portion of the radiation, whereas non-concentrating systems (e.g. flat plate PV) can collect both the direct and diffuse components of sunlight. The direct component of radiation (coming straight from the sun without being scattered or reflected on its way to the collector) makes up the vast majority of sunlight in the equatorial and sunniest locations around the world; but diffuse light (light that has been reflected and scattered on its way to the collector) is a major portion of total sunlight in the more polar and less sunny areas of the world.

Since only direct light can be optically concentrated, concentration requires the ability to track the sun so that the collector is always pointing directly at the sun as it moves across the sky, thus further complicating such systems. However, since solar thermal-electric efficiency benefits greatly from generating higher temperatures to drive the heat engines that convert the thermal energy to electricity, concentrating systems are the standard in this field.

### Solar photovoltaics

At the core of photovoltaic technology is the solar cell, or the material that converts the sunlight to electricity. The physical process behind solar photovoltaics is not within the scope of this article, but suffice it to say that a solar cell is formed at the junction between two semiconductor materials (of which there exists many varieties). Multiple such junctions can be arranged in series (or parallel) that have different abilities to absorb different wavelengths of light (corresponding to different electron band gaps). All of these variations affect how much of the sunlight can be converted to electricity, with the goal being to develop low-cost materials reaching the theoretical limit of efficiency. For a single junction cell this efficiency limit is approx. 30%, but increases to 42% for two-junctions, and 48% for three-junctions, with a theoretical limit of 68% achievable with infinite junctions. Under high concentration the corresponding limits are 40% for a single-junction cell, 55% for two-junctions, 63% for three-junctions, and an 86% theoretical limit with infinite junctions [8].

**Figure 1:**
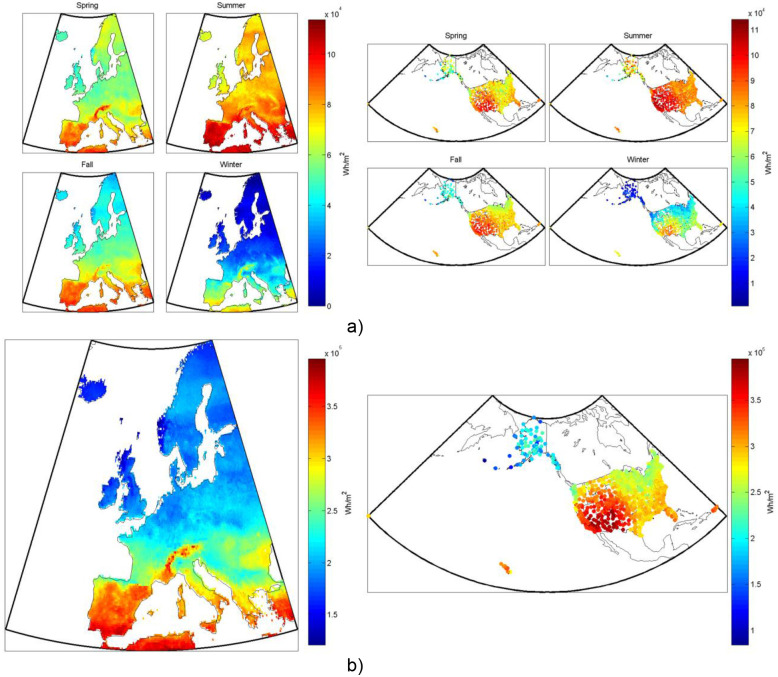
Non-tracking mono-Si PV system’s electricity production, from one square meter of collector, both (a) seasonally and (b) annually in (left) Europe and (right) the US.

A list of the most common solar photovoltaic chemistries used today in order of approximate market share [9] are: polycrystalline silicon (poly-Si), single-crystalline silicon (mono-Si), thin film amorphous silicon (a-Si), thin film cadmium telluride (CdTe), thin film copper indium gallium selenide (CIGS), and multi-junction cells. Silicon technologies are broadly divided into crystalline cells (single or polycrystalline), which make up over 80% of the market, and non-crystalline cells (amorphous). Amorphous cells are generally thin-films, meaning a thin layer of the semiconductor material is deposited on a base layer. This process reduces cost by reducing the amount of material used in the process, but also decreases the efficiency of the cell compared to crystalline silicon cells. CdTe and CIGS cells are examples of non-silicon based commercial thin film technology. At the top end of the technology spectrum, in terms of efficiency, are multi-junction cells, the most advanced of which are generally made up of layers of compounds of group III and V elements on the periodic table. We model several of the most common types (i.e. poly-Si, mono-Si, CdTe, CIGS, and multi-junction) in this analysis, in both fixed tilt and 2d-tracking PV systems. An example of the results for typical annual and seasonal electricity production from a non-tracking mono-Si PV system over Europe and the US is shown in figure 1.

### Concentrating PV

**Figure 2:**
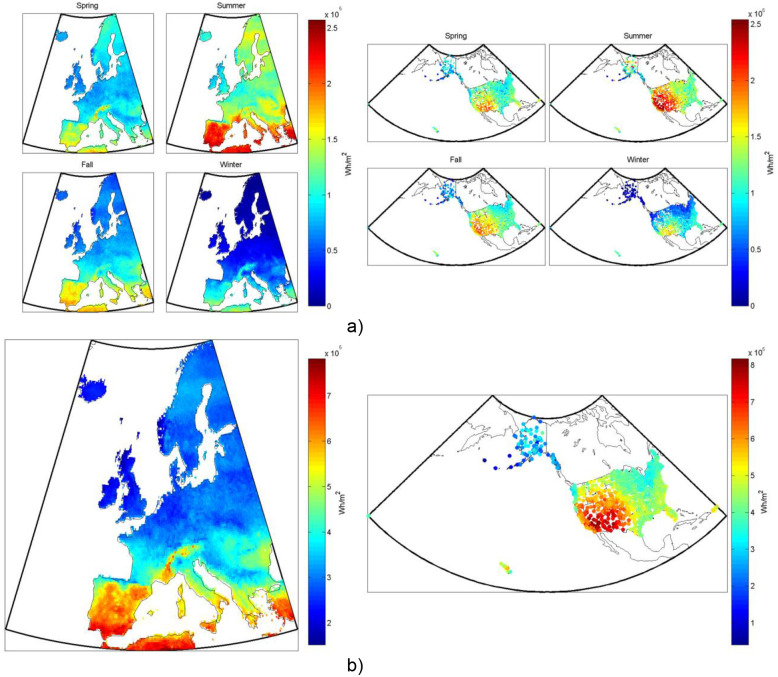
HCPV system’s electricity production, from one square meter of collector, both (a) seasonally and (b) annually in (left) Europe and (right) the US.

In concentrating photovoltaic systems (CPV), the cells are packaged together into a module and usually many modules are mounted on a tracking apparatus where each individual cell is illuminated with highly concentrated sunlight that can be greater than one thousand times as bright as direct sunlight. Commercially, high concentration photovoltaics (HCPV) usually use Fresnel lenses but concentration can also be accomplished with any of the concentrating collector geometries described in the thermal and thermal-electric sections. We model a typical example of an HCPV collector [10] in this analysis using a III–V semiconductor, and show an example of the results in figure 2 as electricity production over Europe and the US for a typical year.

### Solar thermal

At the other end of the solar technology spectrum from photovoltaics is solar thermal technology which collects sunlight and converts the energy to heat. Solar thermal systems use fluids (usually water or a glycol-water mix) to transfer the heat from the collector to a storage tank where it is then used for anything from industrial process heating to domestic hot water and space heating. The main commercialized types of solar thermal systems are those using flat-plate collectors, evacuated tube collectors, and concentrating trough/dish collectors.

Flat plate collectors can be glazed or unglazed. Glazed collectors are insulated on all sides except the glazing (a transparent single or multi-layer) which is facing the sun and allows the sunlight to come in but limits the losses due to convection going out (like a mini greenhouse). The absorber is usually made of copper or aluminum with many channels for the fluid to run through and a selective coating to prevent reflection of the light. Unglazed collectors are often made of plastic polymers, and are usually more appropriate for lower temperature heat demands and warmer climates.

Evacuated tubes are designed like a transparent thermos, where a long cylinder of glass surrounds the channel that the fluid moves through. The space between the glass and the fluid is a near-vacuum to minimize convective losses. The fluid itself is sometimes designed as a heat-pipe allowing for efficient transport of higher temperature fluid to a header where it heats the main circulating fluid in the system. Evacuated tubes also have the benefit of higher acceptance of diffuse light because their cylindrical shape allows collection of light from oblique directions.

**Figure 3:**
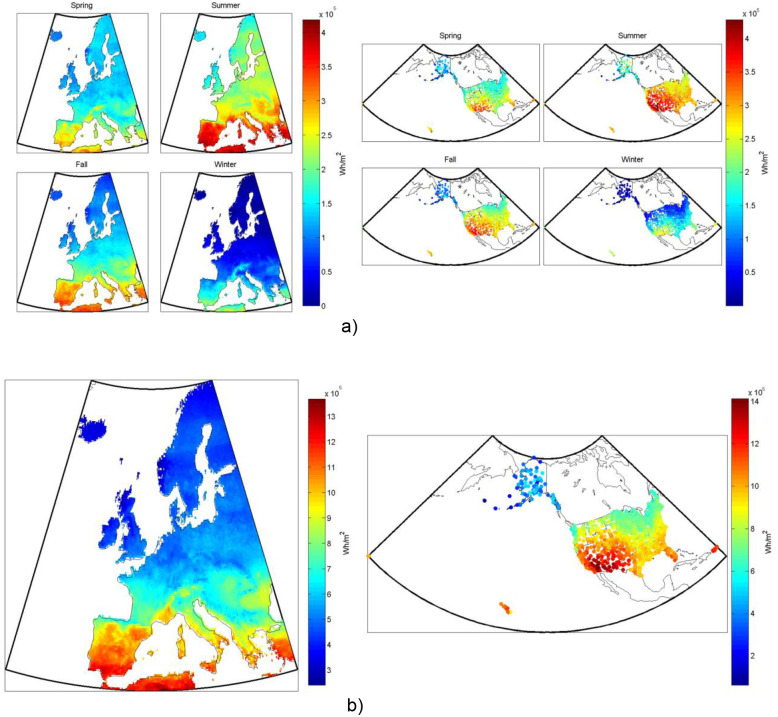
Non-tracking flat-plate thermal system’s heat production, from one square meter of collector, both (a) seasonally and (b) annually in (left) Europe and (right) the US.

Concentrating trough and dish collectors use reflective surfaces in parabolic-like shapes to reflect the sunlight onto an absorber, the main difference between a dish and trough being that a dish is a 3-dimensional parabola (or non-imaging parabola-like shape) whereas a trough is only a parabola in 2-d. Because the incident amount of sunlight per surface area of absorber is higher for a concentrating collector than that for a flat-plate collector and the corresponding thermal losses are lower, due again to the comparatively lower absorber surface area, higher temperatures can usually be obtained with this type of collector than any of the others, especially if the absorber is itself enclosed in an evacuated tube. As they are the main commercialized products for moderate and high temperature solar thermal, we model glazed flat-plate collectors, evacuated tubes, and concentrating troughs in this analysis. An example of the results for typical annual and seasonal heat production from a glazed flat-plate collector is shown in figure 3.

### Solar thermal-electric

Systems that convert sunlight to thermal energy and then to electricity are usually called “concentrating solar power” (CSP) although, as mentioned above, the same concentrating optics could also focus the sunlight on PV cells (CPV) instead of heating a thermal fluid. The scale of CSP systems is usually very large (i.e. power plant), but smaller systems can also be designed, for example, in remote villages for rural electrification. Solar thermal-electric systems offer the advantages of being suitable for operation on other combustible fuels when the sun isn’t shining, and can store energy as thermal energy to later be converted to electricity. This method of storing energy thermally is generally less expensive than storing electricity directly.

To get the high temperatures needed to operate heat engines efficiently, solar thermal-electric systems usually use concentrating solar collectors which can produce fluid temperatures from a couple hundred to over a thousand degrees Celsius. These collector systems can generally be categorized as one of four types: Parabolic trough, linear Fresnel, dish engines, or central receivers. For the purposes of this analysis, only parabolic trough systems are included, although the performance would be comparable to that of a linear Fresnel or dish system based on a Rankine cycle at the same temperatures (500 K max fluid temperature). This moderate temperature allows for simple tracking systems, safe unsupervised operation, and inexpensive plumbing in distributed systems. We exclude central receiver systems and solar Stirling engines from this analysis as they are not well-developed at smaller scale.

The general principle behind solar thermal-electric systems is that a working fluid (usually a molten salt, mineral oil, synthetic heat transfer fluid, or water) is heated to high temperatures at the focus of a concentrating solar collector, and the energy from that hot fluid is then used to run a heat engine. The heat engine is usually based on either a Rankine cycle (the same cycle used in most fossil fuel power plants) or a Stirling cycle.

**Figure 4:**
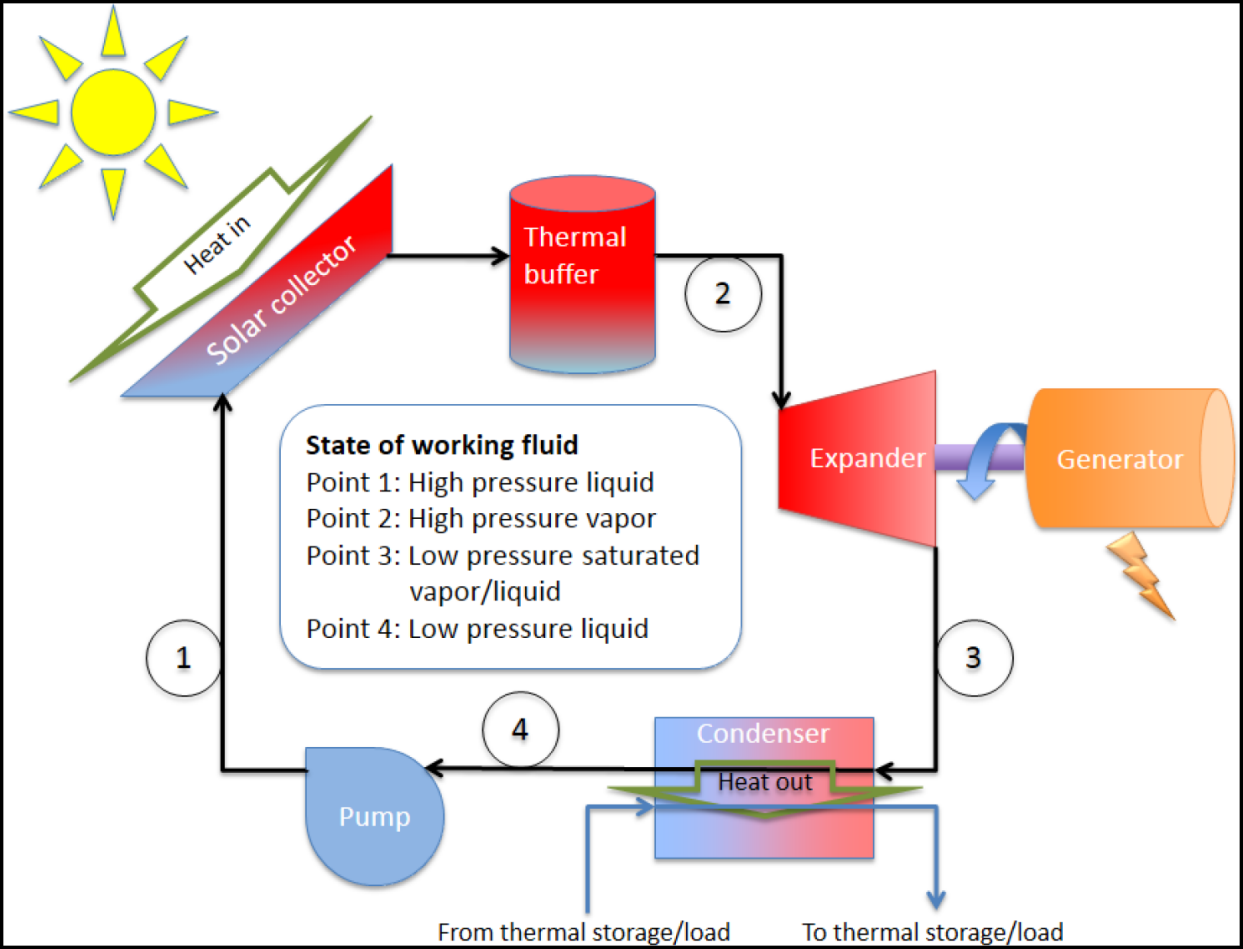
A simple solar CHP Rankine cycle [11].

In a Rankine cycle a fluid (usually water) is compressed, boiled, expanded (where it drops in temperature and pressure in the process of producing mechanical work), and then condensed back to liquid again before starting the cycle over. The mechanical work generated by the expander in this process is converted to electricity by a generator. The schematic of a simple solar Rankine cycle appropriate for distributed heat and electricity generation, as modeled in this analysis, where the heat from the condenser is used for another thermal process (i.e. combined heat and power), is shown in figure 4.

### Hybrid photovoltaic-thermal systems

**Figure 5:**
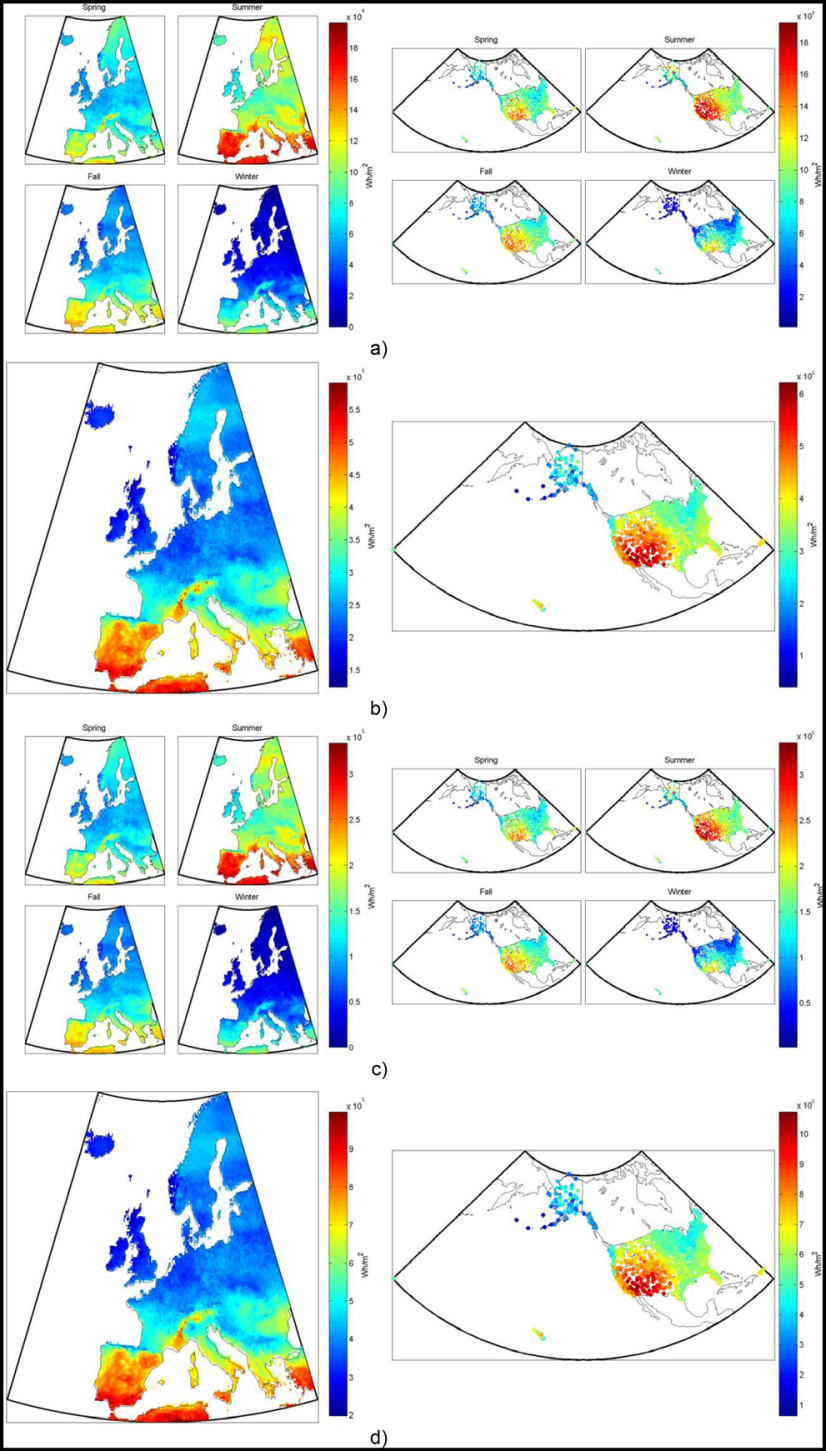
Hybrid CPV/T system’s (a, b) electricity and (c, d) heat production at 373 K, from one square meter of collector, both (a, c) seasonally and (b, d) annually in (left) Europe and (right) the US.

An area of expanding research in the field of solar power is so called hybrid photovoltaic/thermal (hybrid PV/T) systems. These systems combine a thermodynamic heat engine cycle, like in CSP, with a photovoltaic material to boost the overall conversion efficiency of sunlight to electricity. For example, one such system would use an optically selective fluid (e.g. with suspended nanoparticles) running over a photovoltaic material at the focus of a concentrating solar collector (hybrid CPV/T). The fluid would mainly absorb those wavelengths of light that were not useful to the PV, thereby allowing the useful wavelengths to hit the PV, while the other wavelengths heat the thermal fluid to high enough temperatures to run an additional heat engine to produce electricity while also producing “waste” thermal energy from the Rankine cycle (i.e. the same subsystem described in the previous section). The overall solar-electric efficiency from such a system could be higher than either a CSP or PV system alone. We model this technology [12], with thermal and electrical production shown in figure 5.

## Methods

**Figure 6:**
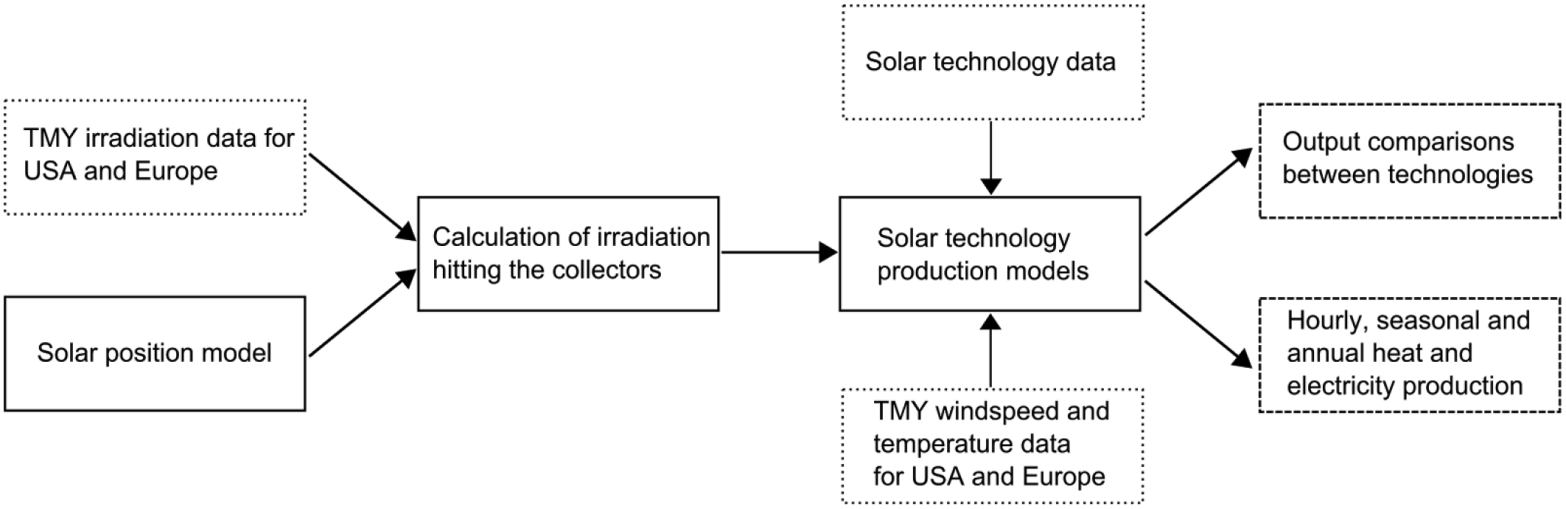
A flowchart of the methodology used for solar modeling.

This research project aims to compare the economic potential of solar technologies across the geographic diversity of Europe and the United States by first quantifying the types and amount of solar resource that each technology can utilize, second estimating the technological performance potential based on that resource, and third comparing the costs of each technology across regions. In this article, we present the first two steps in this process. We use physical and empirically validated models of a total of 8 representative system types: non-tracking photovoltaics, 2d-tracking photovoltaics, high concentration photovoltaics, flat-plate thermal, evacuated tube thermal, concentrating trough thermal, concentrating solar combined heat and power, and hybrid concentrating photovoltaic/thermal. Within the 8 studied system types we model, for comparison, 5 solar-electric, 3 thermal-only, and 2 solar CHP system configurations. These models are integrated into a simulation that uses typical meteorological year weather data (including temperature, irradiance, and wind speed) to create a yearly time series of heat and electricity production for each system over 12,846 locations [13] in Europe and 1,020 locations [14] in the United States. Through this simulation, systems composed of various permutations of collector-types and technologies can be compared geospatially and temporally in terms of their typical production in each location. This methodology is outlined in Figure 6.

We strive to compare each technology based on the closest assumptions possible so that the results of the comparisons are robust without further post-modeling normalization or standardization. To achieve this we use a single solar data source for Europe and another for the US so all points within each of these regions can be compared. The solar position, irradiation and solar technology models are implemented in MATLAB [15], and we look up all thermodynamic fluid properties using NIST software [16]. The actual models for sun position and irradiance are detailed in the sections below, and come from well-referenced sources. The collector technology models vary by type as described below, and in selecting these models we gave preference to empirically verified models for both thermal and PV collectors. The one exception to this is that we use a physical model for the hybrid CPV/T collector developed specifically for this simulation because no appropriate empirically verified model could be found for this type of cutting-edge technology. All other components (e.g. pumps, condensers, inverters, expanders, etc.) in the systems were assumed to have the same efficiency across all system types and under partial load conditions. In all thermal models we ignore thermal storage and assume that the systems can adjust working fluid flow rate to achieve the outlet conditions specified for varying irradiance conditions. We additionally ignore efficiency penalties that could be induced under partial load conditions for expanders and pumps, but do account for irradiance variations’ effect on collector efficiency, and assume naturally that the system will shut down when the irradiance level is so low that the collected energy would be zero (or negative). More detailed assumptions are stated in tables 1, 2 and 3, and in the supplementary information which includes all the code (file S1) as well as additional results graphs (file S2). Nomenclature for all variables and constants in the following equations can be found in table 4.

**Table 1:** Rankine cycle performance constants for solar CHP.

	*T_low_*	*P_low_*	*T_high_*	*P_high_*	*η_Rankine_*	*η_pump_*	*η_expander_*	*η_gen_*	*η_h_*
*Thermal-only system*	300 K	100 kPa	350 K	100 kPa	n/a	0.9	n/a	n/a	0.9
*CHP system*	373 K	100 kPa	500 K	1000 kPa	0.134	0.9	0.8	0.95	0.9

**Table 2:** Thermal collector coefficients.

	*Flat-plate* [21]	*Evacuated tube* [22]	*Concentrating trough* [23]
*a_0_*	0.804	0.718	0.689
*a_1_*	2.564	0.974	0.36
*a_2_*	0.005	0.005	0.0011
*IAM_L_ [0° 10° 20° 30° 40° 50° 60° 70° 90°]*	[1 1 1 0.99 0.97 0.94 0.96 0.72 0]	[1 1 0.99 0.97 0.94 0.87 0.78 0.62 0]	n/a
*IAM_T_ [0° 10° 20° 30° 40° 50° 60° 70° 90°]*	n/a	[1 1.02 1.03 1.04 1.04 1.08 1.17 1.38 0]	n/a

**Table 3:** Selected input parameters for PV technologies.

*Flat plate collector’s rated power per module area (W_p,m2_)*	*(W_p_/m^2^)*
Polycrystalline-Si	149.5
Monocrystalline-Si	200.5
CdTe	125
CIGS	140
***CPV collector’s area per module (A_c_)***	***(m^2^)***
CPV Semprius module	0.3016

**Table 4:** Nomenclature.

Ac	Collector area	[m2]
Ai	Anisotropy index	[-]
AM	Air mass	[-]
a	Coefficient for CPV module temperature	[-]
a_0_–a_2_	Coefficients for thermal collector efficiency	[-]
b	Coefficient for CPV module temperature	[-]
b_0_–b_4_	Coefficients for CPV model	[-]
C	Concentration ratio	[-]
c_1_–c_6_	Coefficients for PV model	[-]
d_0_, d_1_	Coefficients for CPV model	[-]
e	Elementary charge	[C]
E	Power generated	[W m^−2^]
f	Modulating factor for horizon brightening	[-]
G	Solar irradiance collected by the collector	[W m^−2^]
g	Gravitational acceleration	[m s^−2^]
h_0_	Coefficient for PV module temperature	[-]
h	Heat transfer coefficient	[W m^−2^ K^−1^]
I	Irradiance	[W m^−2^]
I_MP0_	Maximum power current at STC	[A]
I_MP_	Maximum power current	[A]
IAM	Incidence angle modifier	[-]
k	Thermal conductivity	[W m^−1^ K^−1^]
k_b_	Boltzmann constant	[J K^−1^]
K	Glazing extinction coefficient	[m^−1^]
L	Glazing thickness	[m]
N_s_	Number of cells	[-]
n	Index of refraction	[-]
n_diode_	Diode quality factor	[-]
P	Watts per installed Watt-peak	[W W_p_^−1^]
q	Heat flux	[W m^−2^]
Rb	Geometric factor	[-]
T	Temperature	[K]
V_MP0_	Maximum power voltage at STC	[V]
V_MP_	Maximum power voltage	[V]
WS	Wind speed	[m s^−1^]
W_p,m2_	Watt-peak per square meter	[W m^−1^]
α	Absorptivity	[-]
α′	Thermal diffusivity	[m^2 ^s^−1^]
α_Imp_	Coefficient for I_mp_ temperature dependence	[K^−1^]
β	Panel tilt from horizon	[°]
β′	Volumetric coefficient of expansion	[K^−1^]
β_Vmp_	Coefficient for V_mp_ temperature dependence	[V K^−1^]
ν	Kinematic viscosity	[m^2 ^s^−1^]
ε	Emissivity	[-]
φ	Latitude	[°]
τ	Transmissivity	[-]
τ_sys_	Transmissivity of all glass and HTF components	[-]
σ	Stefan-Boltzmann constant	[W m^−2^ K^−4^]
δ	Thickness	[m]
δ_tv_	Thermal voltage	[V]
θ	Incidence angle	[°]
η	Efficiency	[-]
ρ	Albedo	[-]
**Subscripts**		
a	Ambient	
b	Beam	
d	Diffuse	
e	Effective	
g	Ground reflected	
g	Global	
o	Extraterrestrial	
H	Horizontal surface	
T	Tilted surface	
n	Normal surface	
cs	Circumsolar	
iso	Isotropic	
hz	Horizon brightening	
in	Inlet temperature of heat transfer fluid	
out	Outlet temperature of heat transfer fluid	
i	Mean temperature of heat transfer fluid	
io	Incident on	
rf	Refraction	
r	Radiation	
c	Convection	
z	Zenith	
ins	Insulation	
inv	Inverter	
gen	Generator	
hx	Heat exchanger	
HTF	Heat transfer fluid	
PV	Photovoltaic	
CPV	Concentrating solar photovoltaic	
Th	Thermal collector	
STC	Standard conditions	
rel	Relative	
mPV	PV module	
mCPV	CPV module	
cell	CPV cell	

### Irradiance model

The total irradiance, *G*, absorbed by a solar collector can be divided into a direct beam component, *I_b,io_*, a diffuse component, *I_d,io_*, and a ground reflected component, *I_g,io_*, as follows:
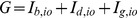
(1)

In the case of a concentrating collector, the diffuse and ground reflected components are assumed to be zero as most concentrating optics will not collect light at oblique angles.

The irradiance absorbed by the collector depends on the orientation of the collector with respect to the sun, atmospheric conditions, and reflection losses due to the light not hitting the collector normal to its plane. For non-tracking collectors we assume an azimuth angle of zero (collector facing due south), and optimize the fixed tilt, β, for yearly production based on latitude, φ, using the correlation by Chang [17] (see appendix for equations).

To account for atmospheric conditions, we calculate irradiance hitting a tilted surface based on the model by Reindl et al. [18]. In addition to the three previously mentioned irradiance components the diffuse component can further be divided into a circumsolar, *I_T,d,cs_*, isotropic, *I_T,d,iso_*, and horizon brightening, *I_T,d,hb_*, components. However, it should be noted that the circumsolar irradiance is diffuse irradiance hitting the collector from the same angle as the beam, and we therefore include it in *I_b,io_*. The angle of incidence modifier (IAM) is used to account for reflection losses. For all tracking collectors, perfect tracking is assumed, so the incidence angle modifier *IAM_b_* is always one, and the other IAMs are zero. Thus the equations for the irradiance components can be written:
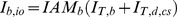
(2)
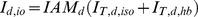
(3)
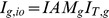
(4)

For detailed equations of the irradiance components see the appendix.

In the case of non-concentrating collectors the angles of incidence for the three irradiance components are not normal to the collector surface most of the day, thus reflection losses need to be accounted for. To quantify these losses, we apply the incidence angle modifier (IAM) to each component. The IAM is the efficiency of a collector at the given incidence angle divided by the efficiency at normal incidence. We calculate the incidence angle for the beam component using the position of the sun and orientation of the panel, and using empirical equations for the diffuse and ground reflected incidence angles [19] (see appendix for equations).

The IAM is different for each collector type; we use a physical model for PV modules and empirical correlations for the thermal collectors when calculating the IAM for each irradiance component (see appendix for details).

### Solar system models

#### Thermal systems

The useful heat, *E_heat_*, and electricity, *E_electricity_*, generated depends on the irradiance hitting the collector and the efficiency of the system’s components, as follows:
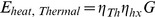
(5)
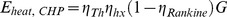
(6)
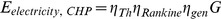
(7)

The thermal-only system efficiency includes the modeled efficiencies for the collector, η*_Th_*, and typical values for the heat exchanger, η*_hx_*, while for the CHP system we also include typical values for the generator efficiency, η*_gen_*, and steam Rankine cycle efficiency, η*_Rankine_*, as calculated from the component efficiencies and working fluid state variables shown in table 1.

The efficiency of the thermal collectors is based on the empirical equation from the EU test standard EN 12975 [20], as follows:
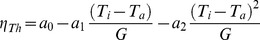
(8)
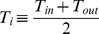
(9)

The mean temperature of the heat transfer fluid, *T_i_*, and coefficients *a_0_–a_2_* depend on the collector and thermal system as shown in tables 1 and 2.

#### PV systems

The electricity produced from PV systems, *E_Electricity,PV_*, depends on the efficiency of the collector and the inverter, as follows:
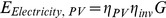
(10)

We assume a constant 95% efficiency for all inverters.

The efficiency of the different PV-technologies, η*_PV_*, depends on the rated peak power per gross area of collector, *W_p,m2_* (see table 3), the share of installed wattage producing power at the given conditions, *P*, and the total irradiance per square meter hitting the collector, *G, as follows:*
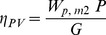
(11)

Details of the PV power equation can be found in the appendix.

#### CPV system

The electricity produced from the CPV system depends on the efficiency of the collector and the inverter, as follows:
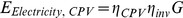
(12)

The efficiency of the CPV module is based on the two-part SAPM model, an empirical model developed by Sandia National Laboratory [10]. The efficiency depends on the current at maximum power, *I_MP_*, the voltage at maximum power, *V_MP_*, at ambient temperature and incident irradiance, and the collector area, *A_c_*, as follows:
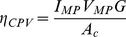
(13)

See the appendix for further details on this model.

#### CPV/T system

The electricity produced from the CPV/T system depends on the combined efficiencies of the CPV and Rankine cycle subsystems. The heat generated is therefore the waste from the Rankine cycle
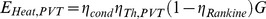
(14)
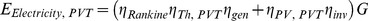
(15)

Prior work in the modeling of concentrating CPV/T systems has resulted in detailed systems of equations to couple together the PV model (which has temperature dependent efficiency) to the thermal model to determine working temperatures of the system [12], [24]–[26]. Such models typically employ transcendental equations for solving for parameters to determine the PV efficiency and contain nonlinear terms with the resulting energy balance equations that contain radiative heat transfer terms. To simplify the prior models developed by Otanicar we have replaced the more complex electrical modeling with a simple temperature dependent efficiency relationship commonly used [27] and shown here:
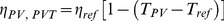
(16)where the reference efficiency, η*_ref_*, is measured at the reference temperature, *T_ref_*, and *T_PV_* is the actual cell temperature. The use of this equation eliminates the integrations and transcendental equations but still leaves the nonlinear terms of the energy balance equations as detailed in the appendix.

## Results

### Solar technology-resource coupling

**Figure 7:**
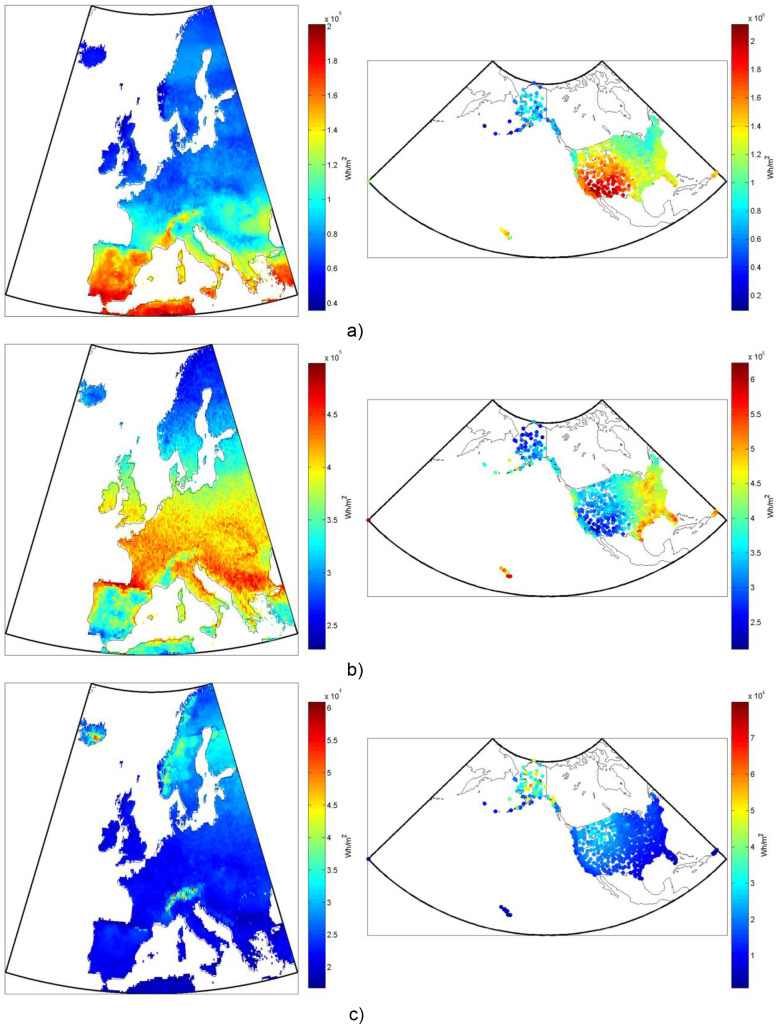
Annual solar irradiance absorbed by one square meter of a non-tracking flat-plate collector tilted at a fixed angle to maximize the yearly total of the three components of radiation: (a) the direct beam and forward scattered circumsolar diffuse component, (b) the non-forward scattered diffuse component, and (c) the ground reflected component. Note that the color scales differ between the subfigures.

**Figure 8:**
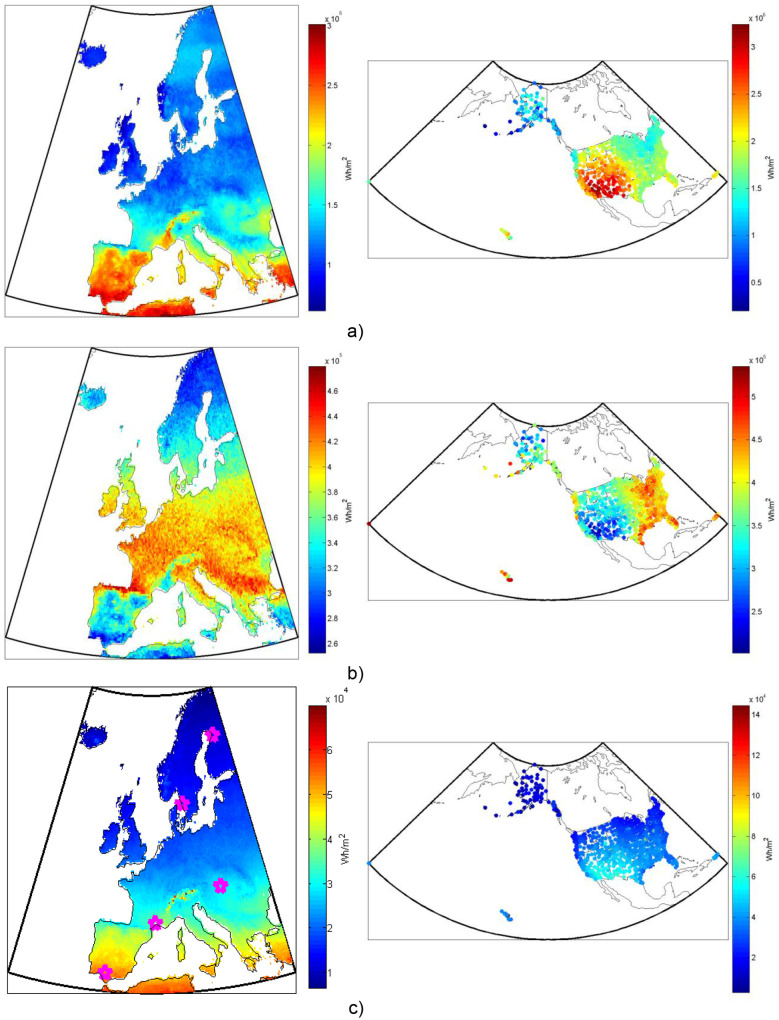
Annual solar irradiance absorbed by one square meter of a 2d-tracking flat-plate collector: (a) the direct beam and forward scattered circumsolar diffuse component, (b) the non-forward scattered diffuse component, and (c) the ground reflected component. Note that the magenta markers indicate the selected European locations referred to in table 5. Note also that the color scales differ between the subfigures.

For comparison, figure 7 shows the three components of irradiance absorbed by a fixed-tilt flat-plate collector (tilt optimized for yearly energy collection), and figure 8 for the same flat-plate collector with 2d-tracking, throughout Europe and the United States. The sum, at each point, of figures 7a, 7b and 7c and figure 8a, 8b and 8c represent the maximum amounts of energy that can be collected from non-tracking and tracking collectors respectively at that location. If a collector is both tracking and concentrating then figure 8a represents the approximate maximum energy collection potential.

Note that although the tracking concentrating collector only uses the beam (and forward scattered) components of the radiation, there is still a substantial increase in the total solar resource utilization possible with concentration in most of Europe and even more so in the United States (i.e. the sum of the beam, diffuse, and ground reflected components incident on a stationary flat-plate collector shown in figure 7 is usually less than the beam component on the tracking collector shown in figure 8). In the clearest areas, including the Alps, Southern Europe, and the Southwestern US the advantage of tracking and concentration is greatest. In the cloudiest and foggiest areas, including the British Isles, most of the central European latitudes between Scandinavia and the Alps, parts of New England, and the Southeastern US, flat-plate collectors have better resource utilization potential.

Just as with thermal systems, there is also a potential, due to the properties of the PV cell material, to increase efficiency and substantially decrease the needed amount of the sometimes expensive photovoltaic material by using concentration. This is typically done using exotic multi-junction high-efficiency solar cells. The economics of concentration with PV is not as favorable as with thermal systems, however, because CPV increases the need for well-managed cooling, tracking and more complex optics, but achieves a smaller increase in efficiency than in thermal systems. Table 5 shows the performance of 10 different PV, solar thermal-electric and thermal-only systems at selected locations both in annual electricity and/or heat production and efficiency as a fraction of the total absorbed irradiance (i.e. the sum of the components shown in figures 7 and 8 respectively for non-concentrating and concentrating technologies). Note that by expressing the efficiency this way one ignores the difference in “collectable” resources between different technology types (e.g. concentrating vs. non-concentrating), so it is perhaps more relevant to compare the total production figures shown.

**Table 5:** Annual electricity and heat production and respective efficiencies (as a percent of total absorbed irradiance) of various solar technologies near several European cities.

	*Sevilla,*		Montpellier,		*Budapest,*		*Göteborg,*		*Oulu,*	
	*España*		France		*Magyarország*		*Sverige*		*Suomi*	
Coordinates:	(37.4°N, −5.9°E)		(43.6°N, 3.9°E)		(47.4°N, 19.1°E)		(57.6°N, 12.1°E)		(65.0°N, 25.5°E)	
	*heat (kWh/%)*	*electric (kWh/%)*	*heat (kWh/%)*	*electric (kWh/%)*	*heat (kWh/%)*	*electric (kWh/%)*	*heat (kWh/%)*	*electric (kWh/%)*	*heat (kWh/%)*	*electric (kWh/%)*
*poly-Si PV (non-tracking)*	0	271/12.5	0	220/12.8	0	175/12.9	0	145/13.2	0	148/13.4
*mono-Si PV (non-tracking)*	0	364/16.7	0	295/17.2	0	235/17.4	0	194/17.7	0	198/18.0
*CdTe PV (non-tracking)*	0	252/11.6	0	204/11.9	0	163/12.0	0	134/12.2	0	135/12.3
*CIGS PV (non-tracking)*	0	259/11.9	0	209/12.2	0	166/12.3	0	137/12.5	0	140/12.7
*HCPV (tracking)*	0	705/25.5	0	502/25.2	0	351/24.8	0	287/24.4	0	325/24.0
*mono-Si PV (tracking)*	0	510/16.5	0	408/17.0	0	320/17.2	0	275/17.7	0	302/18.1
*trough Rankine CHP (tracking)*	1140/41.4	208/7.5	790/39.7	143/7.2	540/38.2	97.9/6.9	433/36.8	78.5/6.7	496/36.6	90/6.7
*hybrid CPV/T (tracking)*	915/33.1	534/19.3	655/32.9	391/19.6	462/32.7	279/19.8	382/32.6	235/20.0	441/32.6	275/20.3
*Flat-plate thermal (non-tracking)*	1270/59.7	0	916/54.7	0	671/50.7	0	492/45.8	0	477/44.4	0
*Evacuated tube thermal (non-tracking)*	1320/59.4	0	982/56.8	0	732/54.4	0	560/51.6	0	558/51.0	0
*Trough thermal (tracking)*	1680/60.6	0	1190/59.9	0	838/59.3	0	689/58.6	0	791/58.4	0

### Electricity production comparison

**Figure 9:**
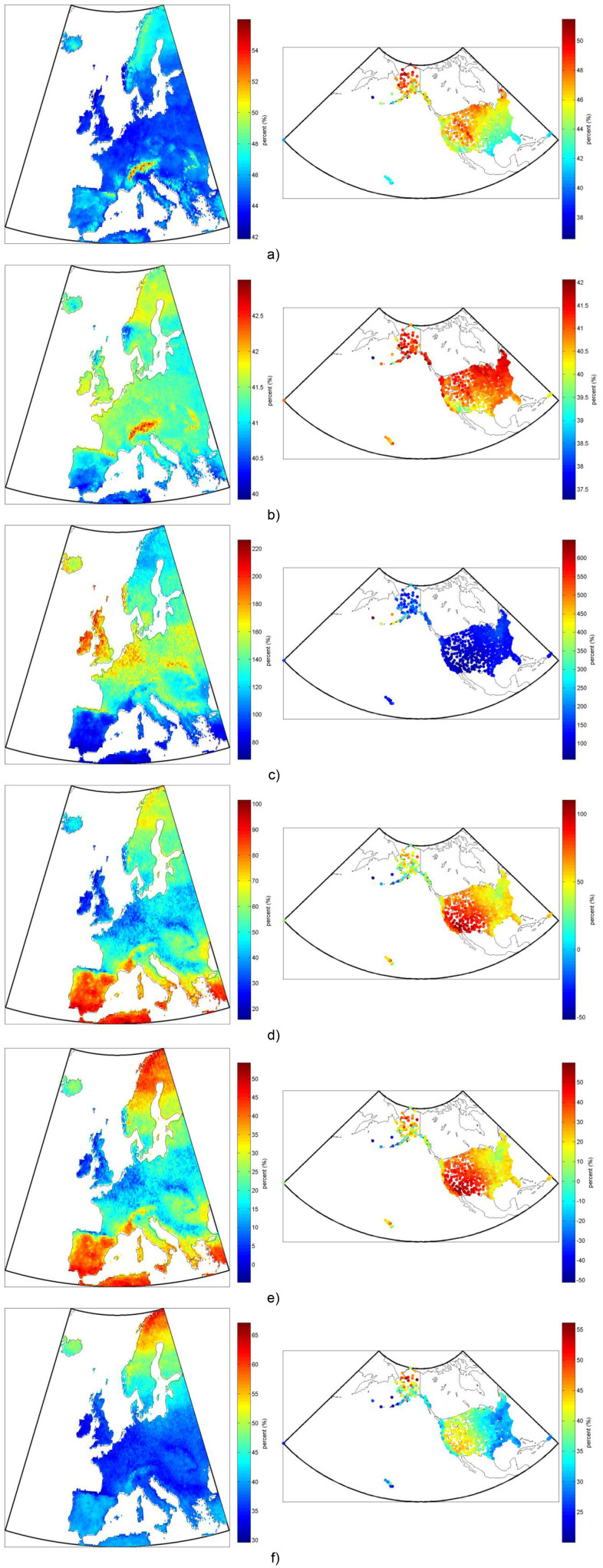
Comparison (in percent) of annual electricity production per square meter of installed collector for several representative solar-electric systems a) non-tracking mono-Si to non-tracking CdTe thin-film b) non-tracking mono-Si to non-tracking CIGS, c) non-tracking mono-Si to solar trough CHP Rankine d) HCPV to non-tracking mono-Si e) hybrid CPV/T to non-tracking mono-Si and f) 2d-tracking mono-Si to non-tracking mono-Si. Note that the reference case is always listed last (e.g. “mono-Si to CdTe” is the mono-Si percent increase or decrease from the CdTe system’s production).

Modeling and comparing the annual production of each of the seven representative solar-electric technology configurations with the same framework across all of Europe and the United States offers some interesting insights. Figure 9a, for example, shows that the relative temperature sensitivity of silicon cells, which exhibit greater performance degradation as cell temperature increases compared to CdTe, gives them a significant advantage (up to 55%) in the colder climatic regions such as in the Alps, Northern Scandinavia, and the Rocky Mountains. This advantage of silicon cells, however, is lessened (to a low of approx. 42%) in comparatively warmer regions of Central Europe, but the relative advantage of silicon increases again (up to 47%) in sunny European regions like Spain, due this time to silicon’s increased gains with higher solar irradiance as compared to CdTe. Figure 9b, comparing mono-Si to CIGS, shows less of these effects as both the temperature and irradiance performance dependence are more similar between the technologies. Furthermore, although the efficiency at *standard temperature and conditions* (STC is 25°C and 1000 W/m^2^) for CIGS is more than 12% greater than CdTe (see Table 3), comparing figures 8b and 8c shows that the *typical* annual production is less than 4% greater in the vast majority of Europe and the US (see also table 5) due to these differences in temperature and irradiance effects.

Comparing mono-Si PV to a thermal-electric steam Rankine cycle at moderate temperatures (500 K, isentropic efficiency of expander of 80%), in figure 9c, shows that PV increases total electric production by at least 50%, but that the greatest increases (of over 200%) are in the cooler areas of lowest direct radiation, including the British Isles, much of the region at latitudes south of Scandinavia and north of the Alps, around the Great Lakes and Alaska.

Comparing CPV/T to flat-plate mono-Si in figure 9e shows the same relative trends, but of course the total production in most locations is greater for the CPV/T technology (−5% to 50%), yet notably CPV/T shows the greatest comparative benefit in the north of Scandinavia, southern Europe, northern Alaska, and the southwestern US. In the north this is due to a combination of a high fraction of direct normal irradiance (DNI) being beneficial for concentrating systems, and low ambient temperatures being beneficial for PV efficiency. In the south, the increased performance of CPV/T is due mainly to the higher fraction of DNI being beneficial for the concentrating system, as compared to flat-plate PV. Additionally, areas that are very cold with extremely overcast weather (like the Aleutian Islands) would suffer lower production with a CPV/T system than with a flat plate mono-Si system for the same reasons.

Figure 9d comparing HCPV to flat plate mono-Si shows that the increased base-efficiency of the multi-junction cell in the HCPV system gives only a 20% increase in total system efficiency in the areas with the lowest fraction of DNI in Europe, but over a 100% increase in total system efficiency in areas with the highest fraction of DNI compared to diffuse irradiance, which occurs in northern Scandinavia, latitudes south of the Alps, and the Southwestern US. Notably again, the Aleutian Islands would actually suffer lower production (-50%) with a HCPV system than a flat plate mono-Si system due to the extreme lack of direct normal radiation due to constant fog.

Finally, figure 9f shows the comparison of a mono-Si PV system with 2d-tracking compared to the same system with fixed-tilt. Notably in this case, as opposed to with the HCPV system, the tracking system is always an improvement over the fixed-tilt system (20–65% more production), with the biggest increases occurring in the northern latitudes, where fixed-tilt systems suffer considerably from the unique path the sun takes across the sky, especially in the summer. Comparing figure 9d to 9f one can see that the concentrating system (HCPV) with high efficiency group III–V photovoltaic cells can still produce 50% more electricity in the areas with the most direct beam radiation compared to a 2d-tracking system with non-concentrating mono-Si cells. However, we can see that in regions with a large percentage of diffuse radiation, tracking non-concentrating PV systems can produce nearly the same amount of power as HCPV systems, even though the latter has the higher efficiency cells.

### Thermal production comparison

**Figure 10:**
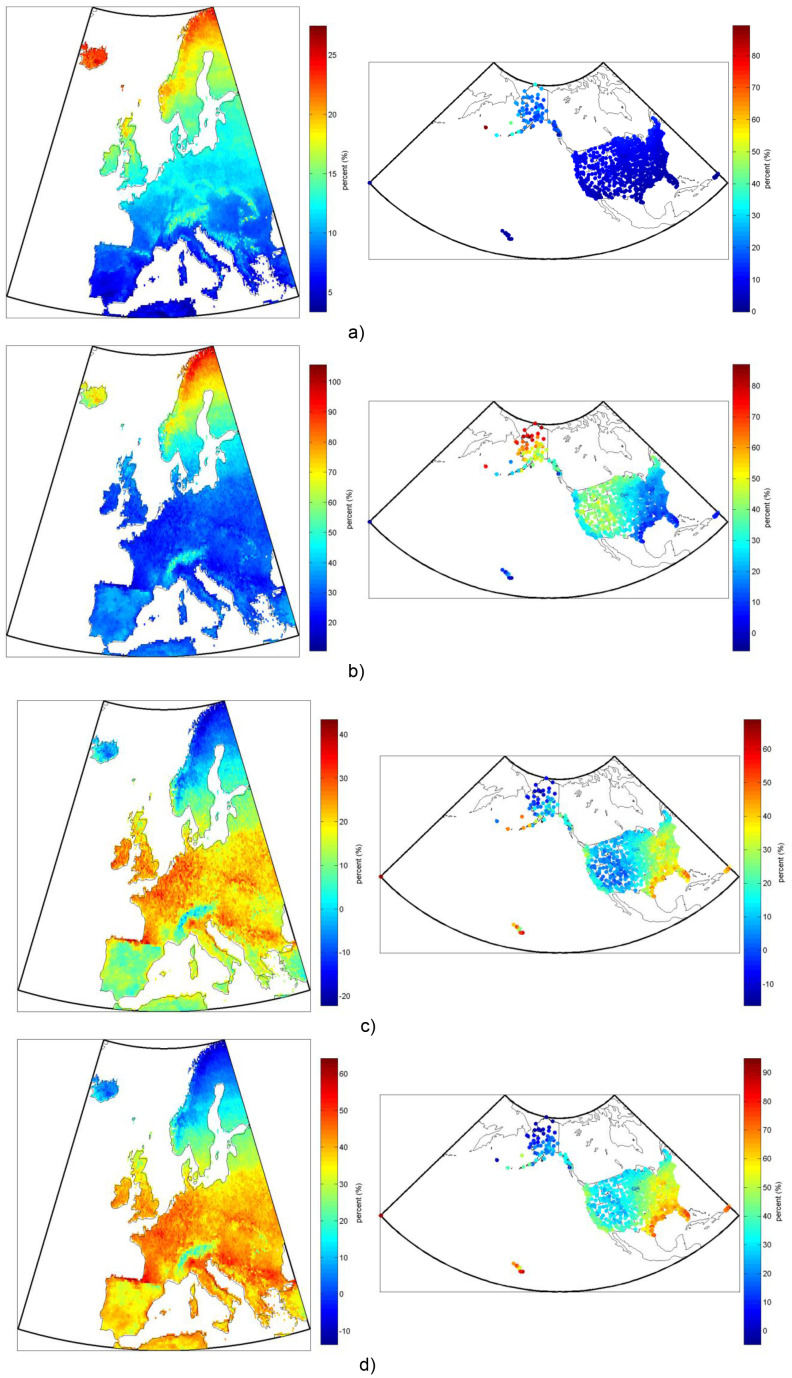
Comparison (in percent) of annual heat production per square meter of installed collector for several representative solar-thermal systems: a) evacuated tube to flat-plate, b) concentrating trough to flat-plate, c) flat-plate to solar trough CHP d) flat-plate to CPV/T. Note that the reference case is always listed last (e.g. “evacuated tube to flat-plate” is the evacuated tube percent increase or decrease from the flat-plate system’s production). Note also that modeled average output temperature from the CPV/T and CHP system is 373K compared to 325K from the thermal-only systems.

In the comparisons between the thermal production of five representative system configurations, the results generally follow the same trends as with thermal-electric systems. Comparison of different thermal collector types, however, offers some new insights. Figure 10a, for example, shows that evacuated tube thermal production exceeds that from flat-plate collectors in all of Europe and the US but is greatest (25% greater in northern Scandinavia, 40%–80% greater in the Aleutian Islands) in the coldest and cloudiest regions, and least (<5%) in the warmest regions (e.g. Southern Spain, Guam, Hawaiian Islands). Clearly the decreased thermal losses of the evacuated tube design seem to give it the biggest advantages, as compared to its increased ability to collect diffuse radiation, as demonstrated by the evacuated tube’s strongest comparative performance in the coldest regions, even those with a lower fraction of DNI.

With the trough thermal system comparison to flat-plate collectors, as shown in figure 10b, the trends show the greatest increase in system production in areas with the highest DNI and coldest temperatures, as would be expected for all concentrating systems. This again is due to the concentrator’s inability to collect any irradiance other than DNI, and the lower thermal losses due to the concentrating absorbers smaller comparative surface area.

Figures 10c and 10d show the thermal output for the thermal-electric systems compared to that of a flat-plate thermal-only system, so in both cases the total heat output of the thermal-electric system is comparatively less because a significant fraction of the thermal energy has been converted to electricity. In fact, comparing figure 10c to 10d shows that the average decrease in heat output of 10–15% of the CPV/T system compared to the solar trough CHP system correlates well with the average doubled relative electrical output of the CPV/T system (i.e. an additional 10–15 percentage points of the collected sunlight is converted to electricity in the CPV/T system, for a total of 20–30% solar-electric conversion).

## Conclusion

Looking at the maximum total irradiance collected by tracking and non-tracking collectors as shown in figures 7 and 8 and comparing that to the total primary energy demand of Europe, which was 2.3*10^16^ Wh in 2011 [28], we can see that depending on region between 120 and 600 times more solar energy can be collected per square meter of collector in the EU-27 than the average current primary energy demand per square meter. For comparison, 5% of the EU land is currently covered by buildings, roads, and artificial areas [29], but using only 0.2% (best solar regions) to 1.0% (worst solar regions) of the land area for solar collectors would collect the same amount of solar irradiance as the entire primary energy demand of the EU-27. This figure is even lower for the US, which has lower average population density and greater average solar resource. Hence, one can conclude that, from a resource perspective, solar energy has the greatest utilizable potential of any renewable technology, but it is also inherently variable, so accurate forecasting and storage will need to be part of any system that utilizes high levels of solar energy.

Additionally, from our production modeling results we conclude that, in terms of both electricity and heat production, the solar technology type can play a large role in the total amount of useful energy that can be collected. Therefore, it is important to consider the regional climate where a system will be installed, instead of comparing technologies based simply on rated power (as is often done). For example, we see that silicon solar cells show a significant advantage in yearly electricity production over thin-film cells in the colder climatic regions, but that advantage is lessened in regions that have high average irradiance. Another result of importance is seen in the northern latitudes, where tracking technologies significantly outperform non-tracking technologies, producing as much as 65% more power with the same collectors. The conclusion is therefore that regional climate differences are, in many cases, of large enough magnitude to shift the most cost-effective technology type from one region to the next.

Continuing work to specify the technology costs in the models developed here will allow us to further understand the market competitiveness of these technologies in comparison to one another, and allow us to apply that information to predict the deployment of each solar technology in future electricity systems, both in comparison to other solar technologies, and to other heat and power production technologies.

## Appendix–Model Description and Equation Reference

### Model of optimum tilt for fixed panels

For latitudes, *φ*, less than or equal to 65°, panel tilt, *β*, is set to:




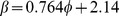




and for latitudes greater than 65°:




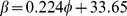




### Model of irradiance components

From Reindl et al. the circumsolar, isotropic and horizon brightening components are dependent on anisotropy index, *Ai*, which is the ratio between the beam normal irradiance and the extraterrestrial normal irradiance. This defines the share of diffuse irradiance that should be treated as circumsolar. The geometrical factor, *Rb*, which affects the amount of circumsolar irradiance, is the ratio of the beam irradiance on a tilted surface to the beam irradiance on a horizontal surface and is equivalent to the ratio of the cosine of angle of incidence to the cosine of the zenith angle. The horizon brightening component also depends on the modulating factor, *f*, and the ground reflected component depends on the albedo, *ρ*, of the installed location. Thus the irradiance model can be written as follows:
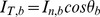

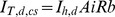

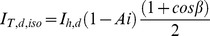

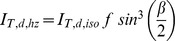

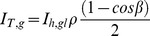

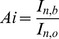

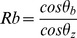

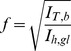


### Empirical model of diffuse and ground reflected incidence angles




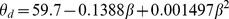




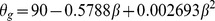




### Models of incidence angle modifiers

In the physical model, used for PV, the IAM is defined as the ratio between the transmittance, τ_0_, at incident angles of zero, and the transmittance at the incident angle for each irradiance component. We calculate the transmittance for each component using the angle of refraction, *θ_rf,b_*, *θ_rf,d_* and *θ_rf,g_* respectively, the glazing extinction coefficient, *K*, and the glazing thickness, *L*, of the module cover, according to the De Soto algorithm with corrections from the PV Performance Modeling Collaborative [30], as follows:
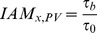

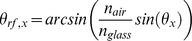

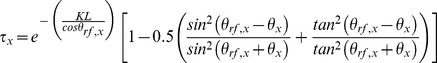


The empirical IAMs used for thermal collectors come from EN 12975 testing certificates for representative collectors of each type. Based on these values, and assuming an IAM of zero at 90° incidence the model performs a linear interpolation to acquire the IAM for the current incidence angle. For tubular collectors, we calculate IAMs in both longitudinal and transverse directions with the incidence angles for diffuse and ground reflected irradiance from Theunissen [31].

### Empirical model of PV module efficiency

The power equation is based on the works of Huld et al. which in turn is a variation of a model put forward by King et al. [3], [32], [33] where *P* depends on the relative irradiance, *G_rel_*, and relative temperature, *T_rel_*, as defined in Huld, as follows:
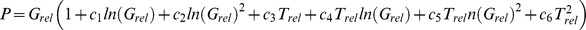

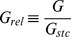

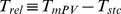

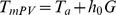


### Empirical model of HCPV module efficiency

The Sandia Semprius HCPV model calculates the actual current, *I_MP_*, and voltage, *V_MP_*, from the current and voltage at maximum power, *I_MP0_* and *V_MP0_*, under standard conditions, and coefficients which describe how the current and voltage change with changing cell temperature, *T_cell_*, and irradiance. We use the Kasten-Young model for calculating air mass, *AM*. The voltage at maximum power also depends on the thermal voltage, *δ_tv_*, and the number of cells in series, *N_s_*, as follows:




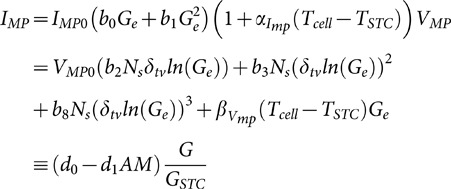




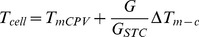




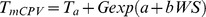




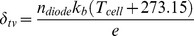




### Physical model of CPV/T module efficiency

The energy balance and heat transfer setup is based on a collector architecture where the working fluid absorbs subgap energy before the PV cell to eliminate waste photons heating the cell (as described in [25]). In order to quickly solve the coupled thermal model (containing nonlinear terms), shown below, we implement a Newton-Raphson methodology for solving nonlinear equations.

**Energy balance equations.**




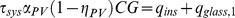




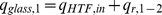




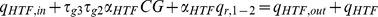




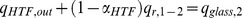




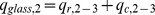




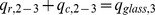




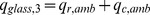




**Heat transfer equations.**
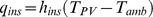

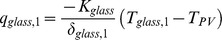

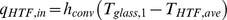

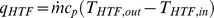

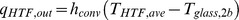

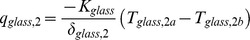

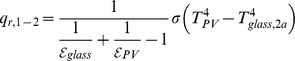

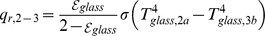

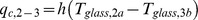

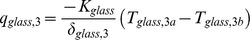

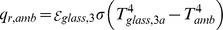

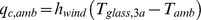
The heat transfer coefficient, 
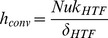
, where Nu = 8.23 (value for constant heat flux between two parallel plates), and 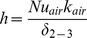, where 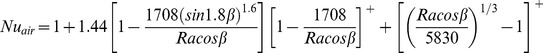, β is the collector tilt (assumed to be zero), and 
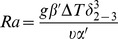
 where ΔT is the temperature difference between the two plates.

## Supporting Information

File S1 Object oriented DCS-CHP model in MATLAB
(RAR)

File S2 Complete set of results graphs.
(RAR)
