# Correction: A Geospatial Comparison of Distributed Solar Heat and Power in Europe and the US

**DOI:** 10.1371/journal.pone.0130242

**Published:** 2015-06-04

**Authors:** The PLOS ONE Staff

There are formatting errors in this paper that were introduced during the typesetting process. The publisher apologizes for these errors.

There is an error in Equation 13. Please view the complete, correct equation here:

$$\eta_{CPV}=\frac{I_{MP}V_{MP}}{A_{c}G}$$

There are errors in the equations in the "Empirical model of HCPV module efficiency" subsection of the Appendix–Model Description and Equation Reference. Please view the complete, correct equations here:

$$I_{MP}=I_{MP0}\left(b_{0}G_{e}+b_{1}G_{e}^{2}\right)\left(1+\alpha_{I_{mp}}\left(T_{cell}-T_{STC}\right)\right)$$

$$V_{MP}=V_{MP0}\left(b_{2}N_{s}\delta_{tv}ln\left(G_{e}\right)\right)+b_{3}N_{s}{\left(\delta_{tv}ln\left(G_{e}\right)\right)}^{2}+b_{8}N_{s}{\left(\delta_{tv}ln\left(G_{e}\right)\right)}^{3}+\beta_{V_{mp}}\left(T_{cell}-T_{STC}\right)$$

$$G_{e}\equiv \left(d_{0}-d_{1}AM\right)\frac{G}{G_{STC}}$$

$$T_{cell}=T_{mCPV}+\frac{G}{G_{STC}}\Delta T_{m-c}$$

$$T_{mCPV}=T_{a}+Gexp\left(a+bWS\right)$$

$$\delta_{tv}=\frac{n_{diode}k_{b}\left(T_{cell}+273.15\right)}{e}$$
