# Correction: A Geospatial Comparison of Distributed Solar Heat and Power in Europe and the US

**DOI:** 10.1371/journal.pone.0264088

**Published:** 2022-02-10

**Authors:** Zack Norwood, Emil Nyholm, Todd Otanicar, Filip Johnsson

## Notice of Republication

An incorrect file was mistakenly included in File S1. This article was republished on February 2, 2022, to correct for this error. Please download this article again to view the correct version.
